# Detection of White Matter Ultrastructural Changes for Amyotrophic Lateral Sclerosis Characterization: A Diagnostic Study from Dti-Derived Data

**DOI:** 10.3390/brainsci10120996

**Published:** 2020-12-16

**Authors:** Fabiola De Marchi, Alessandro Stecco, Zeno Falaschi, Francesco Filippone, Alessio Pasché, Alen Bebeti, Massimiliano Leigheb, Roberto Cantello, Letizia Mazzini

**Affiliations:** 1 ALS Center & Department of Neurology, “Maggiore della Carità” Hospital, University of Piemonte Orientale (UPO), Via Solaroli 17, 28100 Novara, Italy; alen.bebeti@gmail.com (A.B.); roberto.cantello@med.uniupo.it (R.C.); letizia.mazzini@uniupo.it (L.M.); 2 Department of Radiology, “Maggiore della Carità” Hospital, University of Piemonte Orientale (UPO), Via Solaroli 17, 28100 Novara, Italy; alessandro.stecco@uniupo.it (A.S.); zenofalaschi@gmail.com (Z.F.); 20032825@studenti.uniupo.it (F.F.); pascheale@gmail.com (A.P.); 3 Orthopaedics and Traumatology Unit, “Maggiore della Carità” Hospital, Department of Health Sciences, University of Piemonte Orientale (UPO), Via Solaroli 17, 28100 Novara, Italy; massimiliano.leigheb@uniupo.it

**Keywords:** diffusion tensor imaging, amyotrophic lateral sclerosis, magnetic resonance, markers

## Abstract

In amyotrophic lateral sclerosis (ALS), magnetic resonance imaging (MRI) allows investigation at the microstructural level, employing techniques able to reveal white matter changes. In the current study, a diffusion tensor imaging (DTI) analysis, with a collection of apparent diffusion coefficient (ADC) and fractional anisotropy (FA) indexes, was performed in ALS patients to correlate geno- and phenotype features with MRI data, to investigate an in-vivo correlation of different neuropathological patterns. All patients who underwent the MR-DTI analysis were retrospectively recruited. MRI scan was collected within three months from diagnosis. FA and ADC values were collected in corpus callosum (CC), corona radiata (CR), cerebral peduncle (CR), cerebellar peduncle (CbP) and corticospinal tract at posterior limb of internal capsule (CST). DTI analysis performed in the whole ALS cohort revealed significant FA reduction and ADC increase in all selected regions, as widespread changes. Moreover, we observed a higher value of FA in rCR in bulbar patients. A positive correlation between ALS Functional Rating Scale-Revised and FA in rCP was evident. In consideration of the non-invasiveness, the reliability and the easy reproducibility of the method, we believe that brain MRI with DTI analyses may represent a valid tool usable as a diagnostic marker in ALS.

## 1. Introduction

The complex of motor neuron diseases (MNDs) includes a heterogeneous group of conditions, in terms of clinical, genetic and biological aspects. Amyotrophic lateral sclerosis (ALS) is the most common MND, with the involvement of the cortical and spinal motor neurons (MN) [1]. The causative pathogenic mechanisms in ALS remain poorly understood, especially in sporadic cases, but multiple damage mechanisms certainly attack motor neurons and surrounding cells [2]. Currently, no disease-modifying therapy is available for MNDs and all recent experimental trials have failed. Indeed, although several experimental therapies have proven efficacy in animal models, none have been effectively translated to clinical use. The reasons for the repeated failures are multiple and include disease complexity, heterogeneity and lack of biomarkers for patients’ characterization. Substantial clinical heterogeneity between subjects is observed, as the anatomical site of disease onset (bulbar or spinal), degree of upper and lower MN involvement and progression over time. In addition, in roughly 50% of cases, ALS can be associated with cognitive impairment, from moderate executive dysfunctions to a clear form of frontotemporal dementia (FTD), adding variability to functional and motor features [3]. In addition, there are currently no single or multiple biomarkers approved for diagnosis and prognosis. This clearly points out the need for the identification of sensitive markers to promptly differentiate clinical phenotypes and individual risk, useful for patients’ stratification and, consequently, for response optimization to new treatments [4,5]. Post-mortem analyses of ALS brains have indicated that large areas of the white matter (WM), such as the corticospinal tract (CST), the corpus callosum (CC) and fibers in the globus pallidus, the ansa lenticularis and the fasciculus lenticularis, may be involved in ALS [6].

The use of neuroimaging in ALS, including magnetic resonance imaging (MRI) and positron emission tomography (PET), has recently grown rapidly, becoming two well-established technical tools in MNDs to define in vivo markers of underlying pathological alterations [7]. Recent MRI applications to ALS have focused on investigating both at the structural level and at the microstructural level. The structural MRI approach has mainly been used to exclude structural abnormalities; in addition, quantitative structural MRI is able to highlight, on T2-weighted sequences, the CST hyperintensity, described in a high proportion of ALS cases [8]. This technique is also particularly suited to examining long-term structural alterations because of its non-invasiveness and could be useful also for detecting longitudinal changes in a patients’ cohort. MRI allows also investigation at the microstructural level, employing techniques able to reveal WM changes, namely diffusion-weighted imaging (DWI) and diffusion tensor imaging (DTI). DWI is a method providing image contrast based on measurement of the random motion of water molecules [9] and the signal represents an absolute quantification of translational water motion, reflecting the integrity of the brain tissue [10]. DTI is a modality capable of producing quantitative maps of microscopic natural displacements of water molecules that occur in brain tissues as part of the physical diffusion process, allowing for in vivo measurements of white matter fiber orientation [11]. Fractional anisotropy (FA) and apparent diffusion coefficient (ADC) are two of the most used measures of brain WM tract integrity that can be derived from the diffusion tensor imaging DTI dataset. FA reflects damage or sparing of axon fibers in several neurological disorders, showing higher values in highly organized areas and lower values in a tissue where diffusion lacks a specific orientation [12]. The ADC is used to measure the rate and distance of diffusion of water molecules.

The damage of axons and myeline of WM fiber tracts is associated with a decrement of FA values but to an increase in ADC values accordingly.

In the literature, several cross-sectional DTI studies have consistently shown a reduction in FA in the CST in ALS, with a concomitant increase in the ADC values in the same region and in frontal and parietal WM [13,14,15,16]. Nevertheless, the correlations with geno/phenotypic features of ALS patients are not well defined. To our knowledge, only two studies investigated the MRI diffusion imaging in C9Orf72 subjects, observing a regional FA reduction along with the CST, and also in frontal and prefrontal brain areas, compared to SOD1 and sporadic ALS patients, and showing a negative correlation between cognitive and behavioral scores with FA of frontal white matter [17,18].

Similarly, a few studies have employed DTI measures as disease biomarkers: in 2019, Du et al. observed significant direct correlations between the ALS Functional Rating Scale-Revised (ALSFRS-R) scores and FA in bilateral CST, as well as between disease duration and local mean diffusivity in right CST [19]. Similarly, in 2020, Baek et al. reported the DTI values (FA, mean, axial and radial diffusivity), directly correlated with the ALS Functional Rating Scale-Revised (ALSFRS-R) scores and the subgroup analysis revealed more severe and widespread brain degeneration in rapidly progressive ALS, suggesting that DTI findings are useful as imaging biomarkers for evaluating the clinical severity and rate of disease progression [20]. In addition, for neuropsychological characterization, reduction in FA in extramotor associative regions is associated with executive and memory deficits [21]; in addition, frontal and apathy scale appeared to be inversely correlated with FA measures in widespread WM areas [21,22,23].

In the current study, a diffusion analysis, with a collection of ADC and FA indexes, was performed in ALS patients, aimed at investigating the invivo correlation of different neuropathological patterns and, in turn, to correlate geno- and phenotype features, and disease severity as well, with MRI data. In detail, we tried—in a single study—to link the DTI findings as FA and ADC, collected at several levels (including uncommon and less investigated cortico-spinal tracts), both with clinical and genetic data. In addition, all patients underwent a complete neuropsychological evaluation and genetic analysis.

## 2. Materials and Methods

### 2.1. Study Population and Clinical Data Collection

All patients who underwent the MR-DTI analysis from January 2015 to September 2020 at the Tertiary ALS Center at Maggiore della Carità Hospital, Novara, Italy, were retrospectively recruited. Patients’ charts were reviewed retrospectively for all patients included in the study. The study included patients who met El Escorial Criteria for ALS [24], and diagnoses were performed by an experienced ALS neurologist. MRI scan was collected within three months from diagnosis. Patient scans were compared with a control group of healthy persons (selected after a standardized interview by a trained researcher to verify the absence of concomitant diseases) matched for age and sex, performed in the same laboratory. All healthy participants were voluntary. Healthy participants used the same MR equipment and the same pulse sequences protocol (see Section 2.4).

Details of demographics, clinical data including ALSFRS-R [25] for disease severity, force vital capacity percentage (FVC%), disease duration and neuropsychological assessment for all groups were collected at diagnosis and every three months for all disease duration as part of the multidisciplinary care follow-up. The rate of monthly ALSFRS-R progression was calculated using the delta formula: (ALSFRS-R at diagnosis–last ALSFRS-R available)/(last date–first date). A similar analysis was done for delta FVC% progression. Evident brain pathology, including vascular brain alterations and neoplastic lesions, could be excluded by conventional MRI including T1 and T2-weighted and fluid-attenuated inversion recovery sequences.

This study was conducted retrospectively and the patients underwent the MRI scan in our center. As such, patients were exempt from signing consent and our study did not require the approval of the Ethical Committee because we subjected all patients to neuroimaging evaluation as routine in a multidisciplinary approach. No risks were expected for the subjects for the retrospective study design and the results of the study did not have an impact on the diagnosis, prognosis or management of study participants.

### 2.2. Neuropsychological Evaluation

Patients underwent a complete battery of neuropsychological tests in order to evaluate the executive and visuospatial functions, behavior, language and memory. The neuropsychological battery tested global cognition with the Mini Mental State Examination (MMSE), logic and deductive skills with Raven’s Progressive Colored Matrices, executive functions with Cognitive Estimates Test, Frontal Assessment Battery (FAB) and Clock Drawing Test, memory with the Digit Span test and the Short Story Test, attention with the Trail Making A-B Test and Attentive Matrices and language with verbal fluency and comprehension.

Neurobehavioral dysfunctions were determined using the Neuropsychiatric Inventory (NPI). Patients were divided, based on the battery score and Strong criteria published in 2017, into normal cognition, ALS-cognitive dysfunctions (ALS-ci), ALS-behavioral dysfunctions (ALS-bi), ALS-cognitive and behavioral dysfunctions (ALS-ci/bi) and ALS-frontotemporal dementia (ALS-FTD).

### 2.3. Genetic Analysis

All were screened for the main genes causing or associated with amyotrophic lateral sclerosis (SOD1, C9Orf72, FUS, TARDBP). All genes were sequenced with the TruSightTM one Sequencing Panel (Illumina) on a Miseq (Illumina) instrument. C9Orf72 GGGGCC repeat was analyzed also for repeat expansions associated with ALS [26].

### 2.4. MRI Protocol

All MRI scans were performed within three months of diagnosis. MRI scans were obtained with two scanners, a Philips Ingenia 1.5 Tesla and a Philips Ingenia 3.0 T (Philips Healthcare, Eindhoven, The Netherlands). The 1.5 T DTI study protocol comprehended 50 slices, 112 × 110 matrix, with a slice thickness of 2.6 mm, pixel size 2 × 2 mm, the directional resolution was 32 with two b factors (1000 and 0 s/mm^2^); TE and TR were 83 and 6466 ms. The 3.0 T DTI study protocol consisted of 52 slices, 92 × 90 matrix, slice thickness 2.5 mm, pixel size 2.5 × 2.5 mm, the directional resolution was 32 with two b factors (1000 and 0 s/mm^2^); TE and TR were 84 and 3000 ms. The number of scans acquired at each scanner was almost the same between the patients and the healthy group, matched for number of scans at the different scanners, age and sex.

### 2.5. DTI Analysis

DTI quantitative metrics allow us to explore the ultrastructure of tissues, by sampling and measuring water molecule movement (diffusion). The more the tissue is loose and free from structural constraints for water molecule movements, the more the diffusion is “free”. On the contrary, the more the movement is restricted by ultrastructural obstacles, the more the diffusion is “restricted”. Thus, MR imaging measures and reflects the “in vivo probe” of what happens to a tissue. In detail, ADC and FA reflect the interstitial extracellular space width indirectly, by measuring the water molecule movements, and in the brain tissue, this is correlated with axonal density, which is afflicted by ALS pathology: ADC increase and FA lowering reflect in fact a neuronal loss [27].

The analysis of the DTI data was performed using the software Philips IntelliSpace Portal (Best, The Netherlands), using the “MR diffusion” application analyzer. After reconstructing the FA directional map on the basis of the DTI imaging, we proceeded to the evaluation of the corticospinal tract using the “MR FiberTrack” application. Images were evaluated by applying the FA directional map as overlay and the 3D FLAIR sequence as anatomical underlay. The corticospinal tract was reconstructed with a multi-ROI manual tracking, which allowed us to calculate the FA and ADC values in different portions of the motor pathway. FA and ADC values were collected in corpus callosum (CC), right and left corona radiata (rCR) and (lCR), right and left cerebral peduncle (rCP) and (lCR), right and left cerebellar peduncle (rCbP) and (lCbP) and right and left corticospinal tract at posterior limb of internal capsule (rCST) and (lCST).

For the reliability of fiber tracking measurements, two raters experienced in fiber tracking independently performed measurements at each level. Reliability was evaluated using the intraclass correlation coefficients with a score of 0.98 (average measures), and the spatial agreement of tract shape was evaluated with Cohen’s kappa (κ), which found that it was 0.82 (both considered as excellent) [28].

### 2.6. Statistical Analysis

All statistical procedures were carried out with SPSS Version 25.0 (SPSS Inc., Chicago, IL, USA). The two-sample *t*-test was used to examine the between-group (healthy vs. patients) difference in every DTI measurement. We performed comparison analyses in ALS patients based on disease phenotype, cognition, genetic data and progression rate. Univariate analyses were completed using either a chi-squared test (for categorical variables) or independent samples *t*-test (for continuous variables). A comparative analysis between groups was performed using one-way ANOVA for continuous variables and Pearson’s chi-square tests with Bonferroni correction for categorical variables. Survival was calculated from diagnosis to death/tracheostomy or censoring date. The Pearson and Spearman’s rank correlation were used to correlate FA and ADC with clinical features. For each result, a *p*-value of < 0.05 was used to demonstrate a statistically significant difference.

## 3. Results

### 3.1. Patients’ Features

Forty-one ALS patients underwent MRI with DTI and were included in the study. The demographic, clinical and disease progression features are summarized in Table 1. The mean age was 58.53 (SD 12.38) and the mean ALSFRS-R score at diagnosis was 40.18 (SD 5.73). We evaluated the ALSFRS-R scale also considering the single subscores, where we obtained a mean of 10.27/12 (SD 2.04) for bulbar, 9.83/12 (SD 2.15) for upper limb, 7.47/12 (SD 2.70) for lower limb and 11.55/12 (SD 1.15) for respiratory items. Upper motor neuron signs were present in all patients. In addition, a subgroup of 10 patients with the criteria for Upper-MN prevalent phenotype were separately analyzed (Table 1). All patients were undergoing Riluzole treatment when they were recruited.

### 3.2. DTI Findings

**Figure 1:**
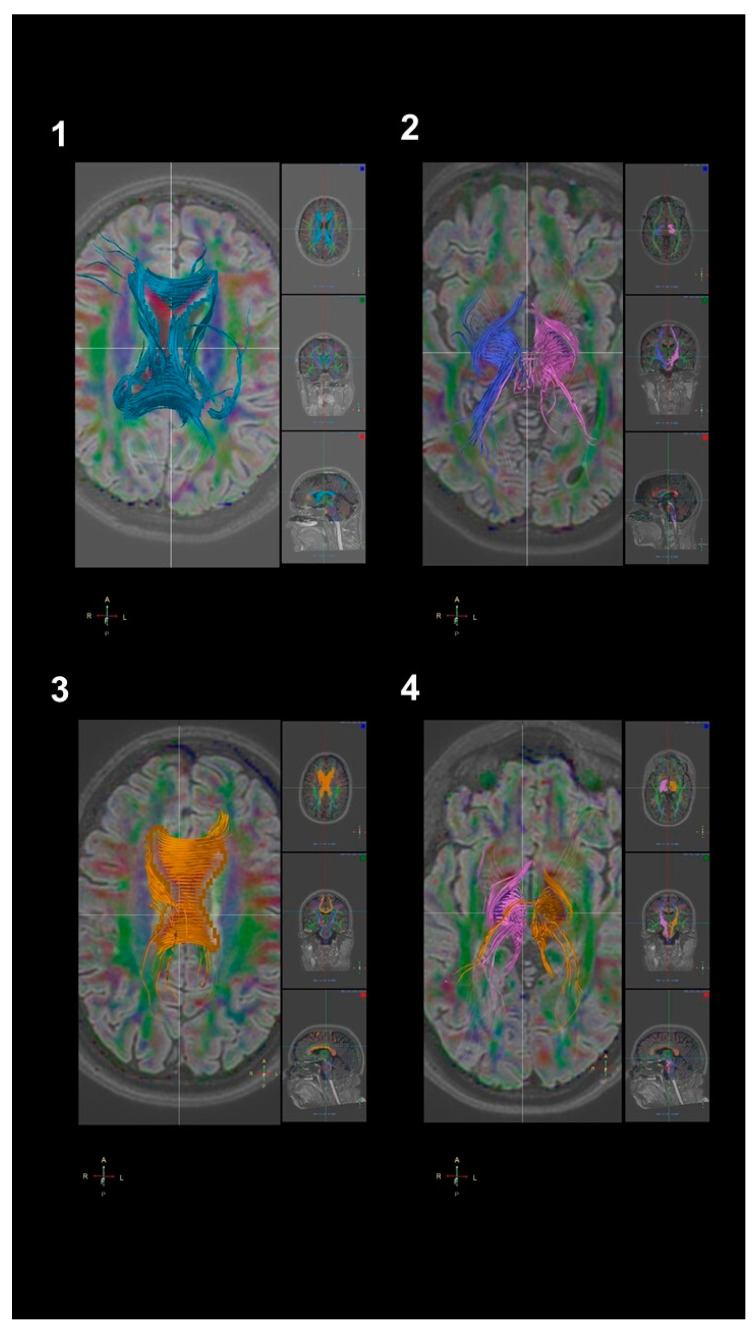
(**1**,**2**) Corpus callosum and corticospinal tract reconstruction in a 44-year-old woman affected by sporadic ALS. (**3**,**4**) Corpus callosum and corticospinal tract reconstruction in a 42-year-old healthy control woman. Images were obtained using the Philips IntelliSpace Portal software, with the fractional anisotropy map as overlay and the 3D FLAIR acquisition as anatomical overlay through single ROI auto-tracking.

DTI analysis performed in the whole ALS cohort revealed significant FA reduction and ADC increase in all selected regions, compared to the healthy control cohort in our Department of Radiology (Figure 1). Indeed, except for the lCR, we observed significant differences between the two groups (*p*-value < 0.05), with diffuse lower values of the FA in patients and, in turn, higher levels of the ADC values. In detail, the FA map revealed the lowest values in the ALS population in the bilateral CST, in the bilateral CR and in the right CbP, while the highest ADC values were detected in the CC and in the bilateral CP (Table 2, first three columns).

### 3.3. Correlation Analysis within Patients

By correlating diffusivity measures in the selected regions and patients’ demographic variables, we observed a direct correlation between age at diagnosis and ADC in the left CP and in the right CR (for both, r = 0.33, *p*-value = 0.03) and a trend for ADC in the left CST (r = 0.28, *p*-value = 0.07). No significant correlations were found with sex. Considering clinical parameters, the ALSFRS-R at baseline was directly correlated with the FA values of the right CbP (r = 0.34, *p*-value = 0.03); in addition, a correlation also between ALSFRS-R and right CP was evident as a trend (r = 0.30, *p*-value = 0.06). In terms of FVC% progression over the disease, downward relations were observed between delta FVC% and the FA values in the right CR and right CbP (respectively, r = −0.45, *p*-value = 0.03 and r = −0.46, *p*-value = 0.02), meaning that a faster decrement in FVC% could be associated with lower FA values in these two regions at baseline. No significant correlations were observed between the FA and the ADC values and the ALSFRS-R subscores. Similarly, no significant correlations were found between diagnostic delay and diffusivity alterations.

### 3.4. Group Comparisons

**Figure 2:**
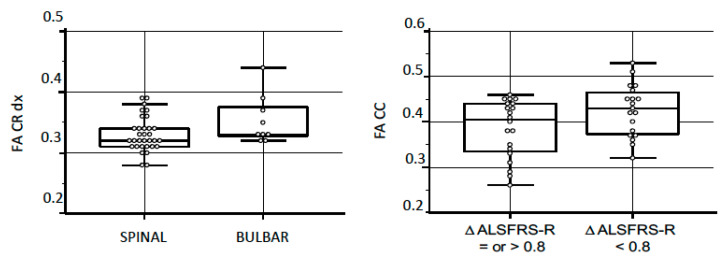
Comparison between clinical features and the FA values. **Left**: the FA values in right corona radiata (based on clinical phenotype); *p*-value: 0.05. **Right**: the FA values in corpus callosum (based on rate of disease progression); *p*-value: 0.04. FA: fractional anisotropy; CR: corona radiata; CC: corpus callosum; ASLFRS-R: Amyotrophic Lateral Sclerosis Functional Rating Scale-Revised.

Disease phenotype. Of 41 ALS patients, 32 had spinal onset and 9 had bulbar onset. The mean ALSFRS-R score at baseline was significantly higher in the bulbar group compared to the spinal group (*p*-value = 0.03) (details in Table 1). No differences were found in age and FVC% at baseline. Table 2 summarizes the FA and ADC values based on disease phenotype. We found significantly higher FA values in the right CR in ALS bulbar patients compared to spinal ALS patients (*p*-value = 0.05) (Figure 2, left). In addition, separating patients with prevalent Upper-Motoneuron (UMN) phenotype, we observed a higher ADC in the right CbP in patients with predominant UMN (*p*-value = 0.01).

### 3.5. Disease Progression

The median of ALSFRS-R rate of progression in our cohort was 0.79/month (SE: 1.80). Consequently, we divided our cohort based on the rate of disease progression in patients above and patients under the cut-off of 0.79/month. Table 1 (last three columns) summarizes the FA and ADC values in the two groups based on disease progression. We observed significantly higher FA values and lower ADC values in the CC of patients with slow progression compared to patients with faster disease progression (respectively, *p*-value = 0.04 for FA and 0.02 for ADC) (Figure 2, right).

### 3.6. Cognitive Profiles and Gene Mutations

Gathering patients both for cognitive profiles (normal cognition and impaired cognition, as ALS-ci, ALS-bi, ALS-FTD) and gene mutations (no mutation, C9Orf72 mutation), we did not observe significant differences in the FA and ADC values between subgroups (data not shown).

## 4. Discussion

In the present study, we evaluated the white matter microarchitecture alterations, measured by the fractional anisotropy and the apparent coefficient diffusion, in a cohort of ALS patients, and the relationships between the diffusivity changes and the geno/phenotypic features of patients.

### 4.1. Differences in the FA and in the ADC between ALS Patients and Normative Group

Overall, we found widespread changes in DTI values in our cohort, with a significant reduction in the FA values measured in CC, bilateral CR, CP, CbP and CST, in ALS patients compared to the normative values acquired within our Department of Radiology. The greatest evidence exhibited a reduction in the FA values in the bilateral CR, extended to the CC, and the bilateral CP, more evident in the right side. In turn, higher values of the ADC were observed in the CC and the bilateral CP. FA reduction in the CR has been reported in previous DTI studies and suggested as an area allowing junctions with the frontal one [29,30]. In turn, as widespread damage, the CC has been described as being altered in [15,31], and this is in line with cardinal histological findings [6]. Though less investigated, also the reduction in the right cerebral peduncle is consistent with a previous study [32], as an extension of damage in the CST. In line with the literature [19,33,34], we also found a reduction in the FA at the corticospinal tract level bilaterally, but more evident on the right, as prevalent expression of upper motor neuron degeneration. This can be explained with the known microstructural right–left asymmetry of the corticospinal tract [35], where higher FA values in the left relative to the right CST have been reported in most healthy right-handed subjects. Hence, we can hypothesize that atrophy of the CST in our patients is more prominent and early in the right CST. In addition, the finding of a higher level of FA in the right CR in ALS bulbar patients compared to spinal ALS patients could be explained by the higher overall ALSFRS-R score at analysis time in the bulbar group and a shorter disease duration.

### 4.2. The Differences in the FA and the ADC Based on Patients’ Functional Level (ALSFRS-R Score)

ALSFRS-R is a functional rating scale commonly used in evaluating patients with ALS. The scale measures the activities of daily living and global function and usually shows a relatively linear decline with disease progression, correlating with physiological measures of progress and with survival [36]. In this study, we found that the FA reduction in the right CP and the right CbP directly correlated with the ALSFRS-R score at baseline. In other words, a lower reduction in the FA values at the right cerebral and cerebellar peduncle is associated with a better functional status at diagnosis. The role of the cerebellum in ALS pathophysiology is not well established. A recent metanalysis describes MRI cerebellar changes, corresponding to cortical or subcortical changes, in ALS [37] and, in addition, some papers on fMRI provided evidence for the precentral degeneration and possible cerebellar compensation in ALS [38,39,40]. Therefore, assuming a possible prognostic role of these values in ALS, we hypothesized a correlation of these and the rate of disease progression. In our patients, those with a rate of ALSFRS-R progression greater than 0.8/month (calculated using the median of progression in this analyzed cohort) had lower values in the FA at the CC, the right CR and the bilateral CST, compared to those with slower progression (although not with a statistically significant difference). However, the absence of a strong correlation between ALSFRS-R progression and the FA value of CST, even if all analyzed patients had a definite diagnosis of ALS, with the upper motoneuron (UMN) involvement, can be related to a low sensibility of ALSFRS-R for the UMN signs and the absence of collection of a specific score for this. In any case, though these results align with what others have published by several authors [20,29,41], more studies examining the correlation of ALSFRS-R monthly progression over time in ALS populations are warranted.

The absence of significant correlations between ALSFRS-R subscores and the FA and the ADC values, in our opinion, does not have great clinical significance in consideration that all patients were at the disease onset and, consequently, the values of ALSFRS-R were most elevated and similar between items.

### 4.3. Lack of Differences in the FA and the ADC Values in c9Orf72 Patients

In our cohort, we did not find any statistically significant differences between the FA and ADC values in sporadic patients and c9Orf72 mutation carriers. Clinically, our C9Orf72 group is relatively younger than the sporadic one; in addition, the c9Orf72 group shows a higher percentage of bulbar patients and higher incidence of cognitive impairment than the sporadic group. These findings are in line with the literature [42]. Similarly, previous reports failed to detect differences between DTI changes in C9Orf72 and sporadic patients. Agosta et al. in 2017 [43] and Muller et al. in 2020 [17] described white matter damage in motor and in frontal and prefrontal brain areas both in sALS and C9Orf72 ALS patients, without differences between groups, but not in the SOD1 group [17]. Floeter et al., in 2018 [18], observed a widespread reduction in the FA in WM of c9Orf72 participants compared to controls, with a frontal predominance. Our findings, in line with papers involving larger samples, support the hypothesis that frontotemporal cortical and widespread white matter involvement is likely to be an effect of the disease progression rather than a C9orf72 signature.

### 4.4. Strengths and Limitations

The main strength of our study is related to an accurate clinical characterization of the patients, both at baseline and during the follow-up, where patients were quarterly evaluated for the entire disease duration. Limitations of this study are, firstly, related to small sample size, particularly the number of familial patients in the absence of other mutations apart from C9Orf72 patients, making a strong DTI differentiation between sporadic and familial patients impossible. Secondly, the absence of a specific scale for UMN involvement may have led to underestimation of the correlation between DTI values and clinical features. In consideration of the non-invasiveness, the reliability and the easy reproducibility of the method, we believe that brain MRI with DTI analyses may represent a valid tool usable as a diagnostic biomarker in motoneuron diseases. However, to achieve this goal, further investigations must be carried out. Future directions should include a larger group of familial patients and longitudinal data in order to enhance the clinical and prognostic significance of our findings.

## Figures and Tables

**Table 1:** Demographic and clinical characteristics of the study participants.

Variables	Patients	Bulbar Onset	Spinal Onset	*p*-Value	UMN-Prevalent	Classic	*p*-Value	ΔALSFRS-R < 0.8	ΔALSFRS-R ≥ 0.8	*p*-Value
Number of patients (*n*)	41	9	32		10	31		20	21	
Males/females, *n* (%)	19 (46%)/ 22 (54%)	3 (33%)/ 6 (67%)	16 (50%)/ 16 (50%)	n.s.	5 (50%)/ 5 (50%)	14 (45%)/ 17 (55%)	n.s.	11 (55%)/ 9 (45%)	8 (38%)/ 13 (62%)	n.s.
Age at onset, mean (SD)	58.53 (12.38)	63.96 (12.10)	56.99 (12.37)	n.s.	50.56 (12.09)	61.09 (11.50)	n.s.	60.81 (11.69)	56.35 (12.90)	n.s.
Diagnostic delay, median (IQR)	10.00 (6–23)	8 (6–24.5)	12 (6–23)	n.s.	14.6 (9–15)	16.70 (11–22)	n.s.	15 (9–18)	9 (6–15)	n.s.
Symptoms onset regions							n.s.			n.s.
- bulbar, *n* (%)	9 (22%)	--	--		2 (20%)	7 (22%)		3 (15%)	6 (28%)	
- spinal, *n* (%)	32 (78%)	--	--		8 (80%)	24 (78%)		17 (85%)	15 (72%)	
ALSFRS-R at diagnosis, mean (SD)	40.18 (5.73)	43.67 (5.27)	39.16 (5.73)	0.03	41.10 (3.38)	39.86 (6.34)	n.s.	39.16 (7.17)	41.10 (4.00)	n.s.
Monthly ΔALSFRS-R, median (IQR)	0.95 (0.45–1.44)	0.95 (0.43–0.95)	0.95 (0.45–1.44)	n.s.	1.50 (0.98)	1.28 (1.99)	n.s.	0.39 (0.26)	2.15 (2.10)	<0.001
FVC% at diagnosis, mean (SD)	86.57 (19.04)	88 (19.59)	86.21 (19.24)	n.s.	99.00 (8.65)	82.22 (19.81)	0.02	80.58 (18.11)	92.22 (18.61)	0.04
Monthly ΔFVC%, median (IQR)	1.44 (1.00–3.32)	1.33 (0.97–1.79)	1.44 (1.00–3.32)	n.s.	2.51 (2.39)	2.94 (5.14)	n.s.	1.27 (1.40)	4.28 (5.73)	0.03
Cognitive function, *n* (%)				n.s.			n.s.			<0.001
- normal	25 (61%)	6 (67%)	19 (59%)		7 (70%)	18 (58%)		8 (40%)	17 (81%)	
- impaired	16 (39%)	3 (33%)	13 (41%)		3 (30%)	13 (42%)		12 (60%)	4 (19%)	
Gene mutation, *n* (%)				n.s.			n.s.			n.s.
- no mutation	34 (83%)	6 (67%)	28 (87%)		9 (90%)	25 (80%)		17 (85%)	17 (81%)	
- C9Orf72	7 (17%)	3 (33%)	4 (13%)		1 (10%)	6 (20%)		3 (15%)	4 (19%)	
Disease duration, median (IQR)	23 (10–42)	23 (12–43.5)	23 (10–42)	n.s.	38 (15–42)	22 (9–25)	0.05	23.5 (5.25–23.75)	21 (6–21.75)	n.s.
Survival rate (alive/deceased), *n*	18 (44%)/ 23 (56%)	4 (44%)/ 5 (56%)	14 (44%)/ 18 (56%)	n.s.	5 (50%)/ 5 (50%)	18 (58%)/ 13 (42%)	n.s.	10 (50%)/ 20 (50%)	8 (38%)/ 21 (62%)	n.s.

SD: standard deviation; ALSFRS-R: Amyotrophic Lateral Sclerosis Functional Rating Scale-Revised; IQR: interquartile range; FVC: force vital capacity; UMN: upper motoneuron; n.s.: not significant.

**Table 2:** Fractional anisotropy and apparent diffusion coefficient values.

Variables	Patients	Controls	*p*-Value	Bulbar Onset	Spinal Onset	*p*-Value	UMN	Classic	*p*-Value	ΔALSFRS-R < 0.8	ΔALSFRS-R ≥ 0.8	*p*-Value
Fractional Anisotropy, mean (SD)												
- corpus callosum	0.40 (0.06)	0.65 (0.04)	<0.01	0.37 (0.07)	0.41 (0.06)	n.s.	0.40 (0.07)	0.40 (0.06)	n.s.	0.41 (0.06)	0.39 (0.07)	0.04
- right corona radiata	0.34 (0.03)	0.61 (0.05)	<0.01	0.35 (0.04)	0.33 (0.03)	0.05	0.32 (0.02)	0.34 (0.03)	n.s.	0.34 (0.03)	0.33 (0.04)	n.s.
- left corona radiata	0.34 (0.04)	0.66 (0.07)	<0.01	0.34 (0.05)	0.34 (0.03)	n.s.	0.33 (0.02)	0.34 (0.04)	n.s.	0.33 (0.03)	0.34 (0.04)	n.s.
- right cerebral peduncle	0.34 (0.04)	0.79 (0.04)	<0.01	0.33 (0.05)	0.34 (0.04)	n.s.	0.33 (0.05)	0.34 (0.04)	n.s.	0.34 (0.04)	0.34 (0.05)	n.s.
- left cerebral peduncle	0.40 (0.05)	0.81 (0.04)	<0.01	0.39 (0.03)	0.40 (0.06)	n.s.	0.40 (0.03)	0.40 (0.06)	n.s.	0.40 (0.06)	0.40 (0.04)	n.s.
- right corticospinal tract	0.53 (0.04)	0.72 (0.08)	<0.01	0.52 (0.02)	0.53 (0.04)	n.s.	0.54 (0.04)	0.53 (0.04)	n.s.	0.54 (0.04)	0.52 (0.04)	n.s.
- left corticospinal tract	0.55 (0.11)	0.75 (0.05)	<0.01	0.52 (0.02)	0.56 (0.13)	n.s.	0.53 (0.03)	0.56 (0.13)	n.s.	0.58 (0.16)	0.52 (0.03)	0.09
- right cerebellar peduncle	0.41 (0.05)	0.66 (0.06)	<0.01	0.40 (0.02)	0.42 (0.06)	n.s.	0.41 (0.04)	0.41 (0.05)	n.s.	0.41 (0.05)	0.41 (0.05)	n.s.
- left cerebellar peduncle	0.43 (0.05)	0.71 (0.10)	<0.01	0.43 (0.01)	0.43 (0.05)	n.s.	0.44 (0.03)	0.43 (0.05)	n.s.	0.44 (0.06)	0.43 (0.03)	n.s.
Apparent diffusion coefficient, mean (SD)												
- corpus callosum	1242 (271)	825 (78)	<0.01	1358 (303)	1210 (258)	n.s.	1252 (291)	1238 (270)	n.s.	1172 (214)	1309 (308)	0.02
- right corona radiata	692 (65)	830 (70)	<0.01	687 (82)	694 (62)	n.s.	697 (82)	691 (60)	n.s.	691 (71)	695 (62)	n.s.
- left corona radiata	764 (120)	739 (75)	n.s.	775 (140)	762 (112)	n.s.	713 (60)	781 (131)	n.s.	792 (136)	740 (102)	n.s.
- right cerebral peduncle	1337 (240)	742 (100)	<0.01	1388 (263)	1323 (236)	n.s.	1429 (274)	1307 (224)	n.s.	1321 (246)	1353 (240)	n.s.
- left cerebral peduncle	1070 (236)	742 (124)	<0.01	1051 (253)	1076 (236)	n.s.	1141 (218)	1047 (240)	n.s.	1057 (218)	1083 (258)	n.s.
- right corticospinal tract	888 (67)	777 (68)	<0.01	898 (48)	886 (72)	n.s.	896 (44)	885 (73)	n.s.	889 (89)	888 (38)	n.s.
- left corticospinal tract	863 (50)	731 (77)	<0.01	861 (26)	865 (55)	n.s.	869 (43)	862 (52)	n.s.	862 (62)	866 (37)	n.s.
- right cerebellar peduncle	965 (243)	729 (133)	<0.01	886 (230)	988 (246)	n.s.	1134 (375)	910 (155)	0.01	919 (148)	1010 (306)	n.s.
- left cerebellar peduncle	791 (134)	745 (69)	n.s.	777 (103)	796 (143)	n.s.	767 (133)	799 (135)	n.s.	805 (137)	779 (134)	n.s.

SD: standard deviation; UMN: upper motoneuron; n.s.: not significant.
